# Symptom Burden and Factors Associated with Acute Respiratory Infections in the First Two Years of Life—Results from the LoewenKIDS Cohort

**DOI:** 10.3390/microorganisms10010111

**Published:** 2022-01-05

**Authors:** Susan Langer, Johannes Horn, Cornelia Gottschick, Bianca Klee, Oliver Purschke, Mahrrouz Caputo, Evelyn Dorendorf, Kristin Maria Meyer-Schlinkmann, Heike Raupach-Rosin, André Karch, Nicole Rübsamen, Mustafa Aydogdu, Matthias Buhles, Frank Dressler, Wolfgang Eberl, Franz Edler von Koch, Torsten Frambach, Heiko Franz, Florian Guthmann, Carlos A. Guzman, Roland Haase, Gesine Hansen, Valerie Heselich, Johannes Hübner, Hans Georg Koch, Carsten Oberhoff, Peggy Riese, Ralf Schild, Sven Seeger, Michael Tchirikov, Stephanie Trittel, Constantin von Kaisenberg, Rafael Mikolajczyk

**Affiliations:** 1 Interdisciplinary Center for Health Sciences, Institute for Medical Epidemiology, Biometrics and Informatics, Medical School of the Martin Luther University Halle-Wittenberg, 06112 Halle (Saale), Germany; Susan.langer@uk-halle.de (S.L.); Johannes.horn@uk-halle.de (J.H.); Bianca.Klee@uk-halle.de (B.K.); Oliver.Purschke@uk-halle.de (O.P.); Rafael.mikolajczyk@uk-halle.de (R.M.); 2 Helmholtz Centre for Infection Research, Epidemiology Research Group Epidemiological and Statistical Methods, 38124 Braunschweig, Germany; mahrrouz_hoodgar@yahoo.de (M.C.); e.dorendorf@hbk-bs.de (E.D.); Kristin.schlinkmann@gmail.com (K.M.M.-S.); heike.rosin@gmail.com (H.R.-R.); 3 Institute of Epidemiology and Social Medicine, University of Münster, 48149 Münster, Germany; akarch@uni-muenster.de (A.K.); Nicole.Ruebsamen@ukmuenster.de (N.R.); 4 Department of Gynecology, Gyneoncology and Senology, Klinikum Bremen-Mitte, 28205 Bremen, Germany; Mustafa.Aydogdu@Klinikum-Bremen-Mitte.de; 5 Department of Gynecology and Obstetrics, Community Hospital Wolfenbuettel, 38302 Wolfenbuettel, Germany; matthias.buhles@klinikum-wolfenbuettel.de; 6 Department of Pediatric Pulmonology, Allergology and Neonatology, Hanover Medical School, 30625 Hanover, Germany; dressler.frank@mh-hannover.de (F.D.); hansen.gesine@mh-hannover.de (G.H.); 7 Department of Paediatrics, Hospital Braunschweig, 38118 Braunschweig, Germany; w.eberl@klinikum-braunschweig.de (W.E.); hg.koch@klinikum-braunschweig.de (H.G.K.); 8 Department of Gynecology and Obstetrics, Hospital Dritter Orden, Munich-Nymphenburg, 80336 Munich, Germany; franz.koch@dritter-orden.de; 9 Department of Gynecology and Obstetrics, Hospital St. Joseph Stift Bremen, 80336 Bremen, Germany; Tframbach@sjs-bremen.de; 10 Department of Gynecology and Obstetrics, Hospital Braunschweig, 38118 Braunschweig, Germany; h.franz@klinikum-braunschweig.de; 11 Department of Neonatology, Children and Youth Hospital AUF DER BULT, 30173 Hanover, Germany; guthmann@hka.de; 12 Helmholtz Centre for Infection Research, Department Vaccinology and Applied Microbiology, 38124 Braunschweig, Germany; Carlos.Guzman@helmholtz-hzi.de (C.A.G.); peggy.riese@helmholtz-hzi.de (P.R.); stephanie.trittel@helmholtz-hzi.de (S.T.); 13 Department of Neonatology and Pediatric Intensive Care, Hospital St. Elisabeth und St. Barbara, 06110 Halle (Saale), Germany; r.haase@krankenhaus-halle-saale.de; 14 Department of Paediatrics, Dr. von Hauner Children’s Hospital, Ludwig-Maximilians-University Munich, 80337 Munich, Germany; valerie.heselich@gmx.net (V.H.); Johannes.huebner@med.uni-muenchen.de (J.H.); 15 Department of Gynecology and Obstetrics, Klinikum Links der Weser, 28277 Bremen, Germany; carsten.oberhoff@klinikum-bremen-ldw.de; 16 Department of Obstetrics and Perinatal Medicine, DIAKOVERE Henriettenstift Hanover, 30559 Hanover, Germany; Ralf.Schild@diakovere.de; 17 Department of Gynecology and Obstetrics, Hospital St. Elisabeth und St. Barbara, 06110 Halle (Saale), Germany; s.seeger@krankenhaus-halle-saale.de; 18 University Clinic and Outpatient Clinic for Obstetrics and Prenatal Medicine, 06120 Halle (Saale), Germany; michael.tchirikov@uk-halle.de; 19 Department of Obstetrics, Gynecology and Reproductive Medicine, Hanover Medical School, 30625 Hanover, Germany; vonkaisenberg.constantin@mh-hannover.de

**Keywords:** birth cohort, respiratory infection, newborn, children, symptom diary, longitudinal observation, infectious diseases, symptom burden, LoewenKIDS

## Abstract

Acute respiratory infections (ARIs) are the most common childhood illnesses worldwide whereby the reported frequency varies widely, often depending on type of assessment. Symptom diaries are a powerful tool to counteract possible under-reporting, particularly of milder infections, and thus offer the possibility to assess the full burden of ARIs. The following analyses are based on symptom diaries from participants of the German birth cohort study LoewenKIDS. Primary analyses included frequencies of ARIs and specific symptoms. Factors, which might be associated with an increased number of ARIs, were identified using the Poisson regression. A subsample of two hundred eighty-eight participants were included. On average, 13.7 ARIs (SD: 5.2 median: 14.0 IQR: 10–17) were reported in the first two years of life with an average duration of 11 days per episode (SD: 5.8, median: 9.7, IQR: 7–14). The median age for the first ARI episode was 91 days (IQR: 57–128, mean: 107, SD: 84.5). Childcare attendance and having siblings were associated with an increased frequency of ARIs, while exclusive breastfeeding for the first three months was associated with less ARIs, compared to exclusive breastfeeding for a longer period. This study provides detailed insight into the symptom burden of ARIs in German infants.

## 1. Introduction

Acute respiratory infections (ARIs) continue to be the most common health problem during childhood worldwide. Although most ARIs are not severe [1], they contribute to a high number of outpatient visits [2], antibiotic prescriptions, hospitalizations [2,3], as well as to socioeconomic burden [4,5] and absenteeism in education and work [6]. In addition, infections with respiratory viruses (e.g., human rhinovirus, enterovirus, and adenovirus) in early childhood can influence the development of chronic and immune-mediated diseases such as asthma, type II diabetes, and obesity later in life [7,8]. Respiratory infections often heal spontaneously, and, in more than 50% of the cases, there is no doctor’s consultation required, and, in even less cases, hospitalization is involved [4,5]. Therefore, it is impossible to determine the true frequency and burden of ARIs based on medical reports or hospital-based studies [9,10]. Individual information on frequency of infections can indicate particularly high susceptibility to infections, and initiate further assessment. For this purpose, contemporary norms are necessary.

Previous observational studies [11,12,13,14] used different assessment methods to determine the frequency of ARI episodes, often including a retrospective assessment. However, retrospective methods may result in under-reporting and recall problems [14], if not only a short time period is considered [15]. It is therefore highly relevant to assess the frequency of ARIs with a real-time approach, such as daily entries into a symptom diary. Symptom diaries are an excellent method to counteract under-recording and allow a detailed description of the burden of disease. Different studies found between three to seven ARIs per year in early childhood (children up to two years old) [16,17,18,19]. In Germany, frequencies of ARIs were last reported for children born in 1990 [1]. Here, only three episodes per year were recorded for children in the first year of life. Since then, no publication was published in Germany which estimated the frequencies of ARIs in children.

There are several factors which might influence the frequency of ARIs in the first two years of life. Previous studies already found out, that older age (compared to the first six months), cold seasons, childcare attendance [18,19], having older siblings, maternal smoking [20], and male sex [21] are associated with a higher number of ARIs, while full breastfeeding [22] is associated with a lower frequency of ARIs. With societal changes, the role of these factors might be changing.

Therefore, we investigated the frequency, the full burden of symptoms, as well as factors associated with ARIs in the first two years of life based on symptom diary data of the German population based on the prospective birth cohort study LoewenKIDS.

## 2. Materials and Methods

### 2.1. Study Population

A detailed description of the study design, methods of recruitment, and data collection is provided elsewhere [14]. Briefly, the LoewenKIDS-study is an ongoing population-based observational birth cohort study, which recruited 782 newborns between November 2014 and February 2018 in five study regions in Germany (Clinicaltrials.Gov Identifier: NCT02654210 (Accessed on: 1 January 2021)). Participants were recruited antenatal and postpartum until the age of three months and are followed up until the age of 15 years. In 2020, all study participants were two years old or older.

### 2.2. Data Collection/Symptom Diary

Parents were invited to keep a daily symptom diary in the first six years of life of their child. They recorded all the child’s symptoms, symptom-free days, doctor consultations, diagnoses, medication, and absence from work or childcare on a daily basis. Participants could choose between a paper-based diary, an online version, or an app. Changes between the different modes were allowed. Symptoms such as fever, wheezing, chills, sore throat, runny/congested nose, increased need to sleep, and increased attachment were included in the symptom diary, as well as severity of the aforementioned symptoms. The symptom diary was developed on the basis of the symptom diary used by the birth cohort ORChID [15] and adapted after a feasibility study [16].

### 2.3. Questionnaires

Parents filled in questionnaires at the birth of their child and at the age of six months, one year, and then annually until the age of 15 years. Questionnaires contain information on social and health characteristics, pregnancy, and birth, as well as on selected diseases and environmental factors.

### 2.4. Classification/Definition of ARI Episode

We adapted the ARI definition proposed by Lambert et al. [23,24,25]. We classified ARIs by distinguishing between A- and B-symptoms. An A-symptom was defined as fever, wheezing, wet cough, and doctor diagnosed pneumonia or otitis media, whereby B-symptoms included dry cough, chills, sore throat, runny or blocked nose, increased need to sleep, loss of appetite, and increased attachment. We defined the beginning of an ARI episode as the occurrence of at least one A-symptom or a day with two B-symptoms. If there were no symptoms for three consecutive days, the episode ended and a new episode could begin. The occurrence of single/isolated B-symptoms were considered within an episode but not as the start of an episode.

### 2.5. Data Processing and Statistical Analyses

Data analysis was performed using R, v. 4.0.5 for Windows. Descriptive analysis included calculating frequencies and duration of ARI episodes by age, sex, and seasonality. Classification into A- and B-symptoms, as well the generation of acute respiratory episodes and the calculation of outcome variables were carried in the R-package lkstaR [26]. Summary statistics are presented as mean (standard deviation, SD) or median (interquartile range, IQR) for continuous variables and frequency (percentage) for categorical variables. We compared different strata according to the number of ARIs using *t*-Test.

ARI frequencies and associations between participant characteristics were estimated using the Poisson regression. Multivariable analysis included duration of exclusive breastfeeding, time of entry in daycare attendance, type of delivery, birth term, sex, and having older siblings. Multivariable models included all the above-mentioned associated factors. Effect estimates and their corresponding 95% confidence intervals (95% CI) are presented. This analysis is based on data collected from 2014 to February 2020.

### 2.6. Ethical Approval

The parents of all children participating in the study provided informed written consent. The respective Ethics Committees of the Martin-Luther-University Halle-Wittenberg, Medizinische Hochschule Hannover and Ludwig-Maximilians-Universität Munich, Germany approved the research protocol.

## 3. Results

### 3.1. Characteristics of Participants

Out of the 782 enrolled children in the LoewenKIDS study, the parents of 732 (93.6%) participants submitted daily symptom diaries. The parents of 433 (55.4%) participants provided entries for 80% of days, however, in order not to miss any potential infection events, we restricted the sample for this analysis to 288 participants (37%), who completed symptom diary on 98% of the days during the first two years of life. The present sample of 288 participants does not differ much in terms of sociodemographic factors from the 732 participants in the overall sample. Characteristics of the study population show that 85% were born at term, 70% spontaneously, 48% were male, 30% had one or more siblings, 85% attended daycare, and 65% were exclusively breastfed for at least four until six months (Table 1).

### 3.2. Symptom Burden

**Figure 1:**
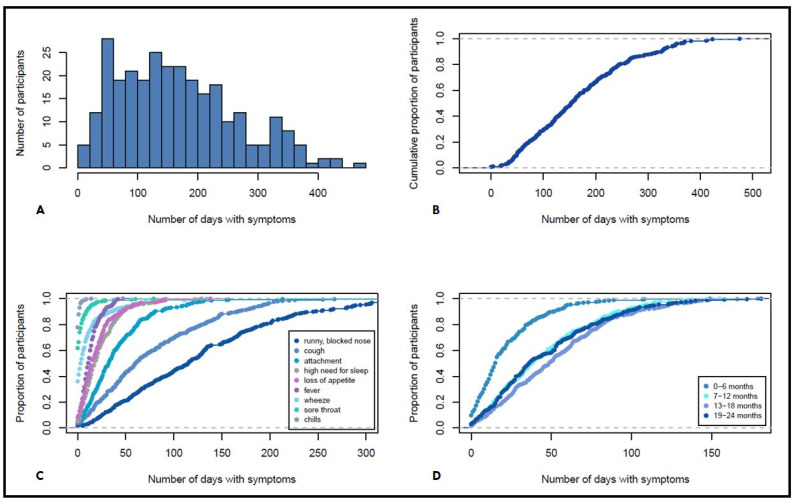
(**A**) Histogram of all days with symptoms within the first two years of life. (**B**) Cumulative distribution of days with symptoms within the first two years of life. (**C**) Cumulative distribution of days with specific symptoms in the first two years of life. (**D**) Cumulative distribution of days with any symptoms in the first two years of life in six months age strata.

In total, 206,001 child-days with diary entries were available for analysis of the included participants. Observed symptoms included cough, wheeze, sore throat, chills, fever, attachment, high need for sleep, loss of appetite, and runny or blocked nose. One or more of these symptoms were reported on 44,441 days (21.6%), corresponding to a mean of 154.3 (IQR: 76.2–216) days with ARI symptom per child (Figure 1A). Symptoms occurred in the first six months of life on average for 19.4 days, at 7–12 months for 41.8 days, at 13–18 months for 49.5 days, and at 19–24 months for 43.4 days (Figure 1B, Table 2).

Figure 1C shows the cumulative distribution of days with specific symptoms in the first two years of life in percentiles. The most common symptoms were runny or blocked nose with an average of 125 days (median: 115.5, IQR: 58–176) and cough in various forms with 76.8 days (median: 60.5; IQR: 30–115). In contrast, rare symptoms such as chills occurred on average 0.5 days (median: 0, IQR: 0), sore throat 3.2 days (median: 0; IQR: 0–3), and wheezing 11.7 days (median: 3.5; IQR: 0–13) on average (Table 2, Figure 1C).

### 3.3. Frequency of Acute Respiratory Infections (ARI) Episodes

**Figure 2:**
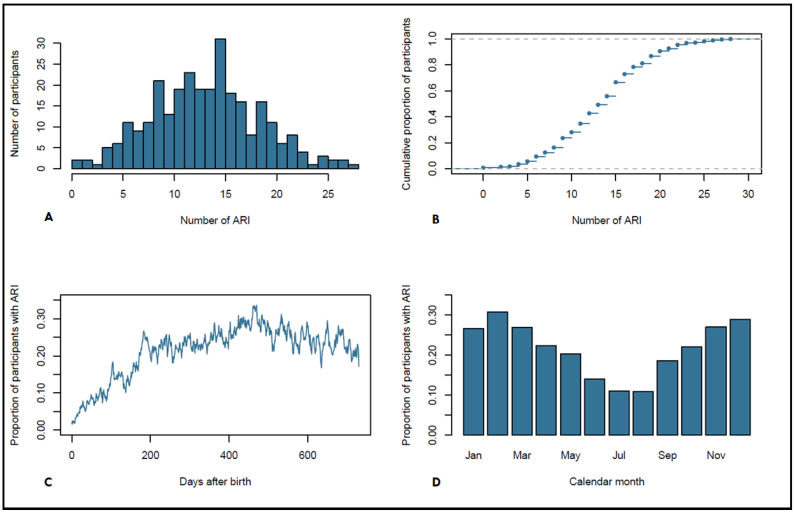
(**A**) ARI episodes of all children in the first two years of life. (**B**) Cumulative distribution of ARI episodes. (**C**) Proportion of children with ARIs in the first two years of life per day (in days after birth). (**D**) Proportion of children with ARIs by month (season).

In the next step, we aggregated the reported symptoms to ARI episodes based on the applied definition. Among the 288 children, a total of 3911 ARIs were reported in the first two years of life (Figure 2A). On average, 13.7 ARI episodes (IQR: 10–17, SD: 5.2, 10th percentile: 7 ARIs, 90th percentile: 20 ARI)) were reported in the first two years of life (Figure 2A). The cumulative distribution of ARI frequency shows that about 25% of children have less than 10 ARI episodes and 25% show more than 17 ARI episodes in the first two years of life independent of sex (Figure 2B). The median age at first ARI episode was 91 days (IQR: 57–128, mean: 107, SD: 84.5) after birth. The mean duration of ARIs was 11 days (SD: 5.8, median 9.7, IQR: 7–14). The proportion of children with ARIs at a given day increased markedly with age (Figure 2C). The frequency of ARI episodes in the first year was slightly lower with a mean of 6.0 ARI episodes compared to the second year with a mean of 7.7 ARI episodes (Table 3).

ARI episodes were more common in the winter months showing a well-known seasonal variation of respiratory tract infections in the northern hemisphere (Figure 2D).

### 3.4. Factors Associated with Acute Respiratory Infections (ARI)

The frequency of ARIs strongly depends on age and seasonality (Figure 2C,D). With increasing age, participants show a marked increase in ARI frequency in the first six months of age (Figure 2C). Furthermore, an increased ARI frequency was observed in winter months compared to the summer months (Figure 2D). In addition, the results from the multivariable analysis in Table 4 show that factors associated with a substantially increased risk of ARIs in the first two years of life are any childcare attendance and having any number of older siblings. However, neither the time point of first childcare attendance nor the exact number of siblings seem to be important for the cumulative number of infection episodes at the age of two. In contrast, the analysis shows that short-term exclusive breastfeeding (less than four months) is associated with a lower risk of ARIs 0.78 [95% CI 0.69; 0.89] within the first two years compared to exclusive breastfeeding for four to six months (Table 4). Children with a longer exclusive breastfeeding of more than six months had the same risk as those with the reference group of four to six months of exclusive breastfeeding. We did not observe any association between birth mode, birth term, or sex of the child.

## 4. Discussion

In the LoewenKIDS birth cohort study, we found that children show an average of 13.7 ARI episodes (first year 6.0 ARIs, second year 7.7 ARIs) with a median duration of 11 days in the first two years of life. ARIs increase with age and occur more frequently during the winter months compared to the summer months. Within the 13- to 18-months lifespan, children in our cohort showed the highest frequency of ARIs, days with symptoms, and occurrence of specific symptoms, such as runny nose or cough during the first two years of life. Attendance at daycare and the presence of siblings in the same household were associated with an increased risk for a higher frequency of ARIs, while exclusive short-term breastfeeding (less than four months) was associated with less ARIs compared to exclusive breastfeeding for four to six months.

The use of symptom diaries to study ARIs is rarely reported. The frequency varies widely between studies, ranging from three to seven ARIs per year in early childhood. Our results are similar to birth cohort studies in Australia [16,18], Scandinavia [27,28], and Canada [29]. However, some studies report lower frequencies such as the Perth study with 4.0 ARIs [17]/4.2 ARIs [5], the Dutch Whistler study with 4.2 [19], or the German Mas-90 Study [1] with the lowest of 3.1 ARIs in the first year of life. It should be noted that all studies took place at different times and under different conditions.

To our knowledge, there is only one study about the frequency of ARIs in early childhood in Germany using symptom diaries, which was published 30 years ago [1]. The Mas-90 study examined children born in 1990 and found an ARI frequency of 3.1 in the first year of life and 3.2 in the second year, which are considerably lower than our results. This is probably because symptoms were recorded only in a kind of symptom diary, rather retrospectively (personal consultation), and additionally, half of the participants with incomplete data were included in the analysis [1]. Therefore, underestimation may be possible. In addition, the number of daycare places for children under three years has increased considerably in Germany since 1990 [30]. It is well known that children attending daycare centers have a higher risk of ARIs [13,18,19,20,31,32], which is in line with the findings of this study. Children in our cohort who entered daycare at the age of 13–26 months show a 1.26-fold risk of developing ARIs (RR: 1.26; CI [1.15; 1.39]) compared with children who did not attend daycare until the age of two years. A cohort study from Pittsburgh [33] as early as 1990 showed that the risk of infection increases with the number of contacts in different care settings. They showed that children in childcare with a group size of at least seven children as well as children with a care time of at least 20 h per week contracted considerably more infections than children who were in home care or in group care with three to six children. Similarly, children having siblings are more likely to have an increased number of infections, which was shown in our cohort as well as in other studies before [1,16,17,20,34]. We could not find any relationship when considering sex, birth mode, or birth term, and we were unable to measure an association between smoking exposure and ARI frequencies because an insufficient number of parents in our cohort reported smoking.

Our cohort showed that short-term exclusive breastfeeding (less than four months) without other nutritional support is associated with a lower risk for ARIs, 0.78 (95% CI 0.69; 0.89), within the first two years compared to exclusive breastfeeding for four to six months. Children with longer exclusive breastfeeding more than six months had the same risk as those with the reference group four to six months of exclusive breastfeeding. There are some studies which showed an association between breastfeeding and a lower risk of ARIs compared to no breastfeeding in early life [20,22,35,36]. However, the results are inconsistent. Cushing et al. showed a protective effect only for lower respiratory infections and no association with upper respiratory infections [22]. Frank et al. showed a protective effect between exclusive breastfeeding in the age period of three to six months compared with no breastfeeding only for ear infections and ARIs with fever and no association for ARIs in general [36]. In contrast, Wright et al. [35] also showed a protective effect of breastfeeding and a reduction in upper respiratory tract infections (URTIs), but only in the first four months of life, and Vissing et al. also showed inconclusive results [20]. On the other hand, there are studies with no evidence for a positive association [37] or even with negative effects for long breastfeeding [1,16,36]. The authors [1,16] assume that the negative effects are due to possible selection effects by, for example, over-reporting of episodes in participating families with higher social status, longer breastfeeding and less smoking.

A birth cohort study from Copenhagen [27] indicated that children in the first year of life had a runny nose an average of 16% of days, 7.9% cough, 4.9% attachment, and 1.5% wheezing. These findings are in line with our results. In comparison to the study from Copenhagen, in our cohort there were slightly more days with runny or blocked nose (17% vs. 16% of days) and a little more cough (10% vs. 7.9% of days). In the younger age strata, we detected only a few days with sore throat and chills. Children at this age cannot yet report sore throat, and the occurrence of chills is certainly also very rare, so we did not expect high frequencies in these age strata in our cohort either.

In 2018, Sarna et al. estimated the average age of the first infection in life at a median of 2.9 months [9]. This is consistent with our estimate of a median of 91 days after birth.

There are many different ARI definitions that have been used in recent research to analyze recorded symptoms [5,16,18,25,27,38], so there is a legitimate question of whether our definition leads to more overestimation or even to underestimation of ARI episodes based on the choice of definition. We adapted the ARI definition proposed by Lambert et al. [25], which in direct comparison of different definitions provided middle estimates in a past study [24].

## 5. Strengths and Limitations

The strengths of this study were the prospective birth cohort study design and the presence of detailed diary data of respiratory symptoms in the first two years of life. Compared with retrospective data, symptom diaries provide more valid data, i.e., higher reporting and incidence rates, thereby mitigating recall bias [39,40,41]. However, the data collection is very time-consuming and challenging. Parents in our cohort were required to keep entries for each day, even if the child was asymptomatic.

It is well known that comparison of ARI episodes is very difficult when different recording methods (retrospective or by physician consultations) have been used. In addition, the studies were conducted in different regions with different environmental factors. However, it must also be said that a comparison is also difficult even with the same recording method if different definitions are used for the identification of an ARI episode based on the symptoms. This is shown by Zoch et al. [24] for six definitions for the identification of an ARI in a single dataset.

Completing a daily symptom diary can be a burden for participants and can affect compliance [42,43] and also leads to tiredness [44]. This is likely the reason why in our study only a subsample of participants submitted complete diaries. We also observed some dropout (11% dropped out of the study in the first two years and even some more stopped recording symptoms). In addition, the symptom diary as a study component likely kept many people from participation.

## 6. Conclusions

This study provides up-to-date, detailed data on the incidence of respiratory diseases in the first two years of life of German children and shows the effects of increasing age, seasonality, daycare attendance, breastfeeding, and the presence of siblings. This study provides pediatricians and researchers with information on the range of infection frequency in generally healthy children. It can be considered as a guideline for the normal occurrence of ARIs in the 21st century. These results show a previously undescribed high frequency and high burden of acute respiratory disease in German children in the first two years of life, which consequently can also represent a great burden for parents and should therefore receive more public attention in this phase.

## Figures and Tables

**Table 1:** Characteristics of 288 LoewenKIDS study participants analyzed in this study. Mean number of ARI episodes and 95% confidence intervals (mean difference) are shown.

Children	Frequency (%) or Mean (±SD)	No. of ARIs * in the First Two Years, Mean	95%CI ^$^ Difference to Reference Group
Sex (*N* = 288)			
Male	139 (48)	13.7	0.2 (−1.00; 1.43)
Female	149 (52)	13.5	Reference
Birth term (*N* = 288) ^§^			
Full-term birth ^~^	266 (92.4)	13.7	Reference
Early-term birth ^~^	22 (7.6)	12.4	−1.3 (−3.18; 0.54)
Birth weight (g) ^§^ (*N* = 286) ^§^	3400 (±488)		
<2500	13 (4.5)	13.1	Reference
>2500–4000	252 (88.2)	13.6	0.5 (−1.91; 2.97)
>4000	21 (7.3)	14.1	1.0 (−2.40; 4.53)
Birth mode (*N* = 287) ^§^			
Vaginal birth	221 (77.0)	13.7	Reference
C-section	66 (23.0)	13.1	−0.6 (−1.96; 0.84)
Number of older siblings (*N* = 286) ^§^			
0	195 (68.2)	13.1	Reference
1	73 (25.5)	14.5	1.4 (0.01; 2.81)
2 or more	18 (6.3)	15.8	2.7 (0.76; 4.76)
Duration of exclusive breastfeeding (*N* = 267) ^§#^			
1 to 3 months ^$^	25 (9.4)	10.8	−3.2 (−5.08; −1.22)
4 to 6 months	165 (61.8)	14.0	Reference
7 to 13 months	62 (23.2)	14.0	0.0 (−1.52; 1.58)
No breastfeeding ^+^	15 (5.6)	12.5	−1.5 (−4.11; 1.13)
Entry in childcare attendance (*N* = 288) ^§^			
>0 to 12 months	98 (34.0)	13.8	2.4 (0.44; 4.42)
13 to 26 months	145 (50.4)	14.1	2.7(0.81; 4.66)
No childcare	45 (15.6)	11.4	Reference
Domestic pets (*N* = 288)			
Yes	80 (27.8)	13.0	−0.8 (−2.16; 0.41)
No	208 (72.2)	13.8	Reference
Parents			
Age mothers at birth in years (*N* = 287)	32.9 (±4.0)	-	-
Age fathers at birth in years (*N* = 285)	35.6 (±5.4)	-	-
Highest academic degree of mothers (*N* = 286) ^§^			
Apprenticeship	77 (27.0)	12.4	−1.5 (−3.02; 0.05)
Bachelor’s degree	16 (6.0)	14.0	0.1 (−2.77; 3.02)
Master’s degree	131 (46.0)	13.9	Reference
PhD/ equivalent	57 (20.0)	14.3	0.4 (−1.06; 1.93)
Other	5 (2.0)	14.8	0.9 (−5.42; 7.27)
Highest academic degree of fathers (*N* = 281) ^§^			
Apprenticeship	86 (31.0)	12.7	0.7 (−2.09; 0.70)
Bachelor’s degree	9 (3.0)	16.7	3.3 (−0.06; 6.68)
Master’s degree	145 (52.0)	13.4	Reference
PhD/ equivalent	37 (13.0)	15.3	1.0 (−0.12; 3.94)
Other	4 (1.0)	18.0	4.6 (−7.40; 16.68)
Monthly household net income in Euro (*N* = 287) ^§^			
<3000	43 (15.0)	13.4	−1.1 (−3.05; 0.86)
3000 to 3999	72 (25.0)	14.5	Reference
4000 to 5000	60 (21.0)	13.1	−1.4 (−3.15; 0.33)
>5000	68 (24.0)	14.1	0.4 (−2.14; 1.31)
Did not provide any information	42 (15.0)	11.8	−2.7 (−4.76; 0.74)
At least one parent with asthma (*N* = 278)	85(30.6)	13.5	0.1 (−1.49; 1.27)
Smoking (*N* = 279) ^§,&^			
Maternal smoking	3 (1)	9.6	-
Paternal smoking	29 (10)	13.2	-

Abbreviations: * ARI means acute respiratory infection; ^§^ participants who filled in the questionnaire, difference to 288 are missing; ^+^ 3 of 36 participants did breastfeed but not exclusively; ^~^ early-term birth (<38 + 4 week), full-term birth (38 + 4–41 + 3 week); ^#^ breast milk exclusively, no other nutritional products; ^&^ sample too small, difference between groups not tested. ^$^ CI: confidence interval.

**Table 2:** Days with symptoms in the first two years of life by six-month lifespans per child and days with specific symptoms.

	0–6 Months	7–12 Months	13–18 Months	19–24 Months	Overall	Cough	Runny Nose	Wheeze	Fever	Attach-ment	High Need for Sleep
Min	0.0	0.0	0.0	0.0	0.0	0.0	0.0	0.0	0.0	0.0	0.0
1st Quantile	4.0	14.8	20.8	16.0	76.3	29.8	57.8	0.0	6.0	16.0	9.0
Median	13	33.0	41.5	33.0	132.5	60.5	115.5	3.50	11.0	33.0	18.0
Mean	19.4	41.8	49.5	43.4	154.3	76.8	125.8	11.7	13.3	41.2	23.1
3rd Quantile	27.3	60.0	71.0	62.0	216.0	115.0	176.0	13.0	18.0	57.3	34.0
Max	149	157	179	183	477.0	363.0	399.0	134.0	47.0	326.0	129.0

**Table 3:** Number of acute respiratory infections (ARIs) in 288 children in the first two years of life in different age groups.

	0–6 Months	7–12 Months	13–18 Months	19–24 Months	0–12 Months	13–24 Months	0–24 Months
Min	0.0	0.0	0.0	0.0	0.0	1.0	2.0
1st Quantile	1.0	2.0	3.0	2.0	4.0	5.0	10.0
Median	2.0	4.0	4.0	3.0	6.0	8.0	14.0
Mean	2.4	3.6	4.0	3.7	6.0	7.7	13.7
3rd Quantile	3.0	5.0	5.0	5.0	8.0	10.0	17.0
Max	9.0	9.0	9.0	11.0	15.0	19.0	28.0

**Table 4:** Multiple Poisson regression analysis of frequency of acute respiratory infections (ARIs) in 288 children during their first two years of life.

Variable	Crude RR ^+^	95% CI	adj. RR *	95% CI
Duration of exclusive breastfeeding ^#^				
No breastfeeding	0.89	(0.77; 1.04)	0.90	(0.77; 1.05)
1 to 3 months	0.77	(0.68; 0.88)	0.78	(0.69; 0.89)
4 to 6 months	1.00	Reference	1.00	Reference
7 to 13 months	1.00	(0.93; 1.08)	1.00	(0.92; 1.08)
Birth mode				
Vaginal birth	1.00	Reference	1.00	Reference
C-section	0.96	(0.89; 1.03)	0.99	(0.92; 1.08)
Birth term				
Full-term birth ^~^	1.00	Reference	1.00	Reference
Early-term birth ^~^	1.11	(0.98; 1.25)	1.10	(0.97; 1.25)
Number of older siblings				
0	1.00	Reference	1.00	Reference
1	1.11	(1.03; 1.20)	1.08	(1.00; 1.16)
2 or more	1.21	(1.07; 1.37)	1.17	(1.03; 1.33)
Entry in daycare				
No daycare in the first two years	1.00	Reference	1.00	Reference
0 to12 months	1.21	(1.01;1.34)	1.27	(1.13; 1.42)
13 to 26 months	1.24	(1.13; 1.37)	1.27	(1.14; 1.42)
Sex				
Male	1.00	Reference	1.00	Reference
Female	1.02	(0.95; 1.08)	1.01	(0.94; 1.07)

^#^ Reference group is a duration of breastfeeding 4 to 6 months breast milk exclusively, no other nutritional products; ^~^ early term birth (<38 + 4 week), full-term birth (>38 + 3 week); ^+^ RR from univariable regression; CI: confidence interval; * RR from multivariable regression are adjusted for factors in Table 4.

## Data Availability

Most of the quantitative results are provided in the tables. Distributions and individual level data in anonymized form can be obtained upon request and from the R-package lkstaR [26].
